# Supplementary material for: The Utilization of Early Outpatient Care for Infants Following NICU Discharge among a National Sample
Source: Children (Basel). 2024 May 4;11(5):550. doi: 10.3390/children11050550 (PMC11119332; doi:10.3390/children11050550)
Supplement: Supplementary file 1 [file children-11-00550-s001.zip › children-2969597-S1.pdf]

Table S1: Marketscan Data Dictionary Codes for Provider Type and Place of Service

	MarketScan Codes
Pediatrician	400
Subspecialty [1]	
Pediatric subspecialist	410-460
Other non-pediatric subspecialists	210-380
Surgeons	500-585
Family practitioner	240
Other MD	
Medical Doctor	200
Osteopathic Medicine	202
Internal Medicine	204
MultiSpecialty Physician Group	206
Non-physician services	
Laboratory	930
Imaging	160, 180, 185, 925
Pharmacy	840 ,935
Physical therapists (or other therapists)	850-855
Supply center	940
Hospice facility	38
Renal dialysis	857
Home health care/nursing	822, 920, 950
Settings where services occurred	
Patient home	12
Office	11
Outpatient Hospital-Off Campus	19
Outpatient Hospital-On Campus	22
Acute care hospital	1
Rural Health Clinic	72
Outpatient not specified	95
Urgent Care Facility /Emergency Room	20, 23
Pharmacy	1, 98
Independent Laboratory	81

[1], Subspecialists includes both pediatric and non-pediatric subspecialists including pulmonology, cardiology, ophthalmology, surgeons, gastroenterology, genetics, otolaryngology, neonatal-perinatal medicine, hematology-oncology, infectious disease, urology
